# Corrosion-Modulating Effect of Pharmaceutical Agents in a Hybrid Coating System on Pure Magnesium

**DOI:** 10.3390/jfb16110406

**Published:** 2025-10-30

**Authors:** Lara Moreno, Adrián Belarra-Rodriguez, Marta Mohedano, Laura Castro, Margarita Chevalier, Raul Arrabal, Endzhe Matykina

**Affiliations:** 1 Departamento de Ingeniería Química y de Materiales, Facultad de Ciencias Químicas, Universidad Complutense de Madrid, 28040 Madrid, Spain; mmohedan@ucm.es (M.M.); lcastror@ucm.es (L.C.); rarrabal@ucm.es (R.A.); e.matykina@quim.ucm.es (E.M.); 2 Materials Science Department, University of Mons, 20 Place du Parc, 7000 Mons, Belgium; 3 Departamento de Radiología, Rehabilitación y Fisioterapia, Facultad de Medicina, Universidad Complutense de Madrid, 28040 Madrid, Spain; abelarra@ucm.es (A.B.-R.); chevalier@med.ucm.es (M.C.); 4 Unidad Asociada al ICTP-CSIC, Instituto de Química Médica (IQM-CSIC), Grupo de Síntesis Orgánica y Bioevaluación, Instituto Pluridisciplinar (UCM), Paseo de Juan XXIII 1, 28040 Madrid, Spain

**Keywords:** magnesium, implants, biodegradable polymer, plasma electrolytic oxidation, corrosion inhibitors, pharmaceutical agents, streptomycin, hybrid coating

## Abstract

There is a knowledge gap about the effect of pharmaceutical agents on the biodegradation of Mg-based resorbable implants. The present work investigates how three common antibiotics and three anti-inflammatory drugs affect the corrosion of high-purity Mg, with and without ceramic and hybrid ceramic/polymeric coatings, using electrochemical impedance spectroscopy and hydrogen evolution tests. A Ca-P-Si-based ceramic coating is developed using plasma electrolytic oxidation (PEO), after the AC voltage and frequency parameters are optimized. A hybrid coating included a PEO and a poly(ε-caprolactone) (PCL) top layer formed by dip coating. High-purity Mg exhibited an instantaneous onset of corrosion with a corrosion rate of 90 μm/year after 24 h of immersion in a modified α-MEM. A hybrid PEO/PCL coating prevents the onset of corrosion for at least 5 h and reduces the H_2_ evolution during the following 90 h by two times by the precipitation of 5–40 μm thick Ca-P surface deposits. Gentamicin, naproxen, streptomycin, ciprofloxacin and paracetamol were found to be corrosion accelerators with respect to bare h.p. Mg, whereas aspirin was found to be an inhibitor. Streptomycin-functionalized PEO/PCL system exhibited an active protection mechanism, triggered upon the release of the coating and substrate cations, associated with the coating defect-blocking action of the insoluble Me(II)-streptomycin chelates.

## 1. Introduction

In recent decades, Mg has emerged as a material for temporary orthopedic implants because it is biocompatible, lightweight and has mechanical properties. It has been shown that pure Mg can stimulate bone regeneration and promote bone tissue growth around the implant because the release of Mg ions promotes cell proliferation and differentiation [1]. However, the degradation rate of pure Mg remains a challenge for temporary orthopedic implants because excessive Mg ions release can cause a local inflammatory response [2], and thus a premature implant failure.

To reduce this inflammatory response and promote proper healing while maintaining an adequate degradation rate, different strategies have been studied, such as the use of bioactive alloying elements, surface modification or pharmaceutical agents [3,4]. However, these techniques have disadvantages when used independently. The use of Mg alloys requires careful consideration of biocompatibility issues arising from the release of ions from alloying elements (e.g., aluminum and rare earths), which may accumulate in surrounding tissues and induce cytotoxic, neurotoxic, or hepatotoxic effects [5,6]. In this context, high-purity magnesium (h.p. Mg) has emerged as a promising and safer alternative, as it minimizes the risk of adverse biological responses while maintaining sufficient degradation control. The main advantage of pure Mg over Mg alloys lies in the absence of secondary phases, which prevents galvanic coupling and subsequent localized corrosion, thereby slowing down the degradation process. Additionally, pure Mg lacks alloying elements that could be toxic or harmful to the human body, but it has the disadvantage of lower mechanical properties compared to certain magnesium alloys [7]. Recent studies have shown that high-purity Mg suture anchors exhibit a suitable degradation rate and maintain mechanical integrity for up to 12 weeks post-implantation, providing reliable fixation without inducing toxic effects in animal tissues or organs [8,9]. Among the impurities, iron (Fe) has the key effect on the corrosion rate of pure Mg [10,11], and its content should be kept as low as possible.

Another way to improve the properties of pure Mg is through the use of protective coatings. In recent years, various synthetic biodegradable polymers have been investigated as anti-corrosion coatings, such as poly (lactic acid) (PLA), poly(ε-caprolactone) (PCL), poly (1,3-trimethylene carbonate) (PTMC), poly(etherimide) (PEI), etc. [12,13]. However, polymer coatings can detach from the substrate relatively easily because the connection between them is only a physical bond formed by the immersion method [14]. It should also be noted that a smooth substrate surface is usually not conducive to adhesion of polymer coatings, a rough substrate surface being preferable [15].

Plasma electrolytic oxidation (PEO), employed as a pretreatment method, effectively solves the polymer-adhesion problem [16,17]. An increasing number of publications have focused on hybrid systems that combine PEO coatings with layers of biodegradable polymers, such as poly(ε-caprolactone) (PCL) and poly (lactic acid) (PLA), to achieve more effective protection [18,19,20]. These polymer–ceramic systems have been shown to enhance the mechanical stability and durability of Mg implants in physiological media, while also acting as platforms for controlled drug delivery. The incorporation of pharmaceutical agents, including antibiotics, anti-inflammatory compounds, and osteoinductive factors, into polymer coatings represents a significant advancement in orthopedic implants, targeting tissue regeneration support and infection risk reduction [21,22]. Some works have demonstrated their performance in vitro, supporting their translational potential [23,24]. However, drug incorporation introduces a complex and often poorly controlled variable in corrosion studies.

A large number of studies have demonstrated that pharmaceuticals commonly prescribed by healthcare professionals, including antibiotics and non-steroidal anti-inflammatory drugs (NSAIDs), can interact with metallic surfaces and influence their corrosion behaviour through mechanisms such as complexation with metal ions, local pH shifts, or disruption of protective surface oxides [25,26,27]. While these interactions have been investigated in various materials, including stainless steel, bronze, and bare magnesium alloys (AZ31 or AZ61), their effect on multilayer systems [27,28,29,30], particularly on hybrid ceramic-polymer coatings, remains insufficiently studied. As demonstrated in previous research by the authors, the incorporation of drugs such as ciprofloxacin and paracetamol into polymer–ceramic coatings on Mg-Zn-Ca alloys has been explored [24,31]. These previous studies have demonstrated the efficacy of drug incorporation and its improvement in corrosion protection of these hybrid coating systems under controlled conditions. However, the influence of clinically relevant pharmaceutical agents, administered as part of actual therapeutic treatments, on the long-term performance of these systems remains unexplored. This gap is especially critical in clinical practice, where drug selection is driven by the medical needs of the patient rather than the compatibility of the implant material. Consequently, implants may be exposed to pharmaceuticals that actively accelerate corrosion. Therefore, from a biomaterials engineering point of view, it is more pertinent to focus on corrosion investigation of more aggressive pharmacological agents than on ideal corrosion inhibitors in order to probe the true limits of a hybrid coating’s protective capability.

In this context, the present study aims to bridge the gap between the design of surface engineering and realistic pharmacological exposure. This will be achieved by evaluating a representative hybrid system composed of a plasma electrolytic oxidation (PEO) layer sealed with a biodegradable poly(ε-caprolactone) (PCL) top layer. A selection of clinically relevant antibiotics and anti-inflammatory drugs frequently prescribed during postoperative recovery was used to simulate realistic chemical challenges. This approach enables the first systematic investigation of the corrosion-modulating effects of pharmaceutical products on high-purity, surface-functionalised magnesium coatings, allowing their robustness to be assessed and their degradation mechanisms to be elucidated. The results offer valuable insights for the rational design of multifunctional coatings that can maintain structural and chemical stability in complex pharmacological environments.

## 2. Materials and Methods

### 2.1. Materials

The Institute of Surface Science of Helmholtz-Zentrum Hereon (Geesthacht, Germany) supplied a cast ingot of h.p. Mg with a nominal composition illustrated in Table 1. The ingot was cut into square-shaped specimens with dimensions of 30 × 23 × 2 mm^3^ and 15 × 15 × 3 mm^3^ and ground successively down to P1200 grit emery finish.

Poly(ε-caprolactone) (PCL) was provided by Natureplast (Mondeville, France) [32]. All solvents were procured from Merck and utilized in their original state, without undergoing any additional purification processes.

The anti-inflammatory agents employed in this study were aspirin (acetylsalicylic acid) with 99% purity and paracetamol (4-acetaminophenol) with 98% purity, both of which were supplied by ACROS Organics^TM^ (Thermo Fisher Scientific, Geel, Belgium). Fisher Scientific supplied Naproxen, a non-steroidal anti-inflammatory drug, which was characterized by analysis to be 99% pure. The antibiotic agents, namely ciprofloxacin (98% purity), gentamicin sulphate (99% purity), and streptomycin sulphate (99% purity), were provided by ACROS Organics^TM^.

### 2.2. Plasma Electrolytic Oxidation (PEO) Treatment

The PEO process was conducted in a 2 L jacketed electrochemical cell for a duration of 300 s. The experimental setup involved the utilization of a 50% duty cycle square AC waveform, accompanied by an initial ramp and a limiting current density of 100 mA/cm^2^. The process was powered by an AC supply, model EAC-S2000, provided by ET Systems electronic. The parameters of the process are displayed in Table 2. The concentrations of alkaline electrolytes are as follows: Na_3_PO_4_·12H_2_O: 10 g/L, Na_2_SiO_3_·5H_2_O: 9 g/L, KOH: 1 g/L, CaO: 2.9 g/L; pH: 13.68 and σ: 16.52 mS/cm, which were prepared using deionized water. The counter electrode was composed of a cylindrical mesh constructed from 316 stainless steel. The experimental setup involved the use of a Keithley KUSB-3116 data acquisition card, which was employed to electronically capture the RMS voltage and current responses at a sampling rate of 0.1 s. Subsequently to the surface treatment, the samples were washed in deionized water and dried using warm air.

### 2.3. Polymer Coating

The poly(ε-caprolactone) (PCL) was dissolved in chloroform (50 mg/mL), and the Mg/PEO sample was immersed in the solution using an automatic dip coater machine (Nadetech ND-DC, Noain, Navarra, Spain, with software dipper step motor controller) using 0.3 mm/s of withdrawal speed and 2 cycles of immersion. After this, the sample was removed from the solution and dried at room temperature. For the incorporation of the drug into the polymer-coated layer, 5 wt% of the drug with respect to the polymer weight was dissolved in a PCL/chloroform solution.

### 2.4. Surface Characterization

Images of the samples in plan view and cross-section, conducted to evaluate the PEO treatment and the polymer coating, were obtained using JSM-6400 equipment (JEOL Ltd., Tokyo, Japan) at 20 kV, which features an OXFORD LINK PENTAFET 6506 EDS analysis system (Oxford Instruments, Abingdon, UK) along with a backscattered electron detector (BSE). The specimens’ cross-sections were prepared for observation by grinding to a P1200 finish, then polished successively with 3 and 1 μm diamond pastes and coated with graphite.

The phase composition of the PEO-coated sample was evaluated using Philips X’Pert X-ray diffractometer (Malvern Panalytical, Almelo, The Netherlands) operated in grazing incidence under the following conditions: CuK_α_ = 0.154056 nm, scan speed 0.04°/s and range 2Ɵ: 10–90°. The specimen’s spectra were examined using X’Pert HighScore software v5.3a (Malvern Panalytical, Almelo, The Netherlands) in combination with the ICDD PDF-4+ database (ICDD, Newtown Square, PA, USA) for phase identification.

The InfiniteFocusSL optical profilometer from Alicona GmbH (Graz, Austria), equipped with a ×50 magnification lens, was utilized to assess the roughness (S_a_, arithmetical mean height of the area). The topography outcomes were obtained utilizing the IF-MeasureSuite software v5.1 (Alicona GmbH, Graz, Austria).

A micro CT system featuring an X-ray source (L10951-04, Hamamatsu Photonics, Hamamatsu, Japan) and a flat panel detector with a pixel size of 50 µm (C7940DK-02, Hamamatsu Photonics, Hamamatsu, Japan) was utilized to characterize uncoated and PEO-coated h.p. Mg following 4 days of exposure in the H_2_ test. Two scans were conducted at a voltage of 65 kV, a tube current of 90 μA and an exposure duration of 10 s. These were performed with minimum horsepower. With the Mg substrate sample, 1000 projections were collected with the sample placed 8 cm from the X-ray source and at 105 cm from the detector (M = 14x). The size of the pixels in the reconstructed volume slices was 3.49 µm. For the sample coated with PEO, 1500 projections were obtained with the sample positioned 8 cm from the source and 64 cm from the detector (M = 9x). The size of the pixels in the reconstructed volume slices was 5.5 µm.

The FTA1000 Drop Shape Analysis System (First Ten Angstroms, Portsmouth, VA, USA), along with the FT32 software v2.4 (First Ten Angstroms, Portsmouth, VA, USA), was utilized to assess the wettability of the materials. The drops were documented in a trigger mode within 25 s from the instant the droplet was within 1 mm of the surface. Contact angles were assessed over 50 frames captured at 0.5 s intervals, averaging three measurements for each surface treatment; measurement variability was within ±10%.

### 2.5. Electrochemical Impedance Spectroscopy (EIS)

The electrochemical assessments were conducted at 37 °C in 125 mL of modified, organic-free α-MEM solution (Minimum Essential Medium Eagle-alpha; 6.8 g/L NaCl, 2.2 g/L NaHCO_3_, 0.4 g/L KCl, 0.12 g/L Na_2_HPO_4_, 0.09 g/L MgCl_2_·6H_2_O and 0.2 g/L CaCl_2_). The potentiostat used was the GillAC (ACM Instruments, Cumbria, UK). The experiment employed a conventional three-electrode configuration, comprising a graphite counter electrode, an Ag/AgCl-3M KCl reference electrode, and the sample functioning as the working electrode (area ~12 cm^2^). Electrical impedance spectroscopy (EIS) measurements were conducted over a maximum immersion time of 24 h, utilizing a sinusoidal signal with an amplitude of 10 mV (versus OCP) and a frequency range from 100 kHz to 10 mHz. Data were fitted using ZView software v3.5c (Scribner Associates Inc., Southern Pines, NC, USA).

To evaluate the corrosion behaviour of the samples, α-MEM was selected as the test medium because it provides a chemically defined and reproducible environment that minimizes interference from complex organic components typically present in complete culture media. This choice facilitates accurate interpretation of electrochemical data, such as EIS and hydrogen evolution, while maintaining physiological relevance. In contrast, conventional media such as DMEM contain amino acids, vitamins, and proteins that can unpredictably interact with Mg surfaces and alter corrosion kinetics. Therefore, simplified formulations like α-MEM are preferred for mechanistic studies of biodegradable metals in simulated biological fluids [33].

### 2.6. Hydrogen Evolution Experiment

Hydrogen evolution tests were conducted in order to assess the degradation rate of Mg, PEO coating, and PCL/PEO samples following a period of 4 days of immersion in α-MEM solution. The samples, with an exposed area of 12 cm^2^, were positioned in 25 mL burettes inside a 9 L plastic chamber. The pH of the physiological solution was consistently maintained at 7.4 by regulating the flow of carbon dioxide (CO_2_) via a switch that was connected to a pH sensor. This system was consistent with the methods outlined in our earlier research [34].

### 2.7. Selection of the Pharmaceutical Agents

The selection of pharmaceutical agents was carried out by hydrogen evolution test (without pH control and room temperature) using a non-coated substrate at room temperature in inorganic α-MEM during 4 days of immersion, using 1 cm^2^ area. 30 mg of the drug was dissolved in 250 mL of inorganic α-MEM with a final concentration of 120 μg/mL. The H_2_ gas released was collected by inserting a 25 mL inverted burette into the cell, and the volume was noted at 15 min intervals during the first few hours and at regular intervals during the following days until the end of the test.

### 2.8. Ion Release Analysis

The release of Mg ions was evaluated using a using a Perkin Elmer Avio 220 Max ICP-OES (PerkinElmer Inc., Waltham, MA, USA) fitted with a cyclonic spray chamber (Glass Expansion, Melbourne, Australia). Argon was utilized as the carrier gas to ensure the maintenance of the plasma. The operational conditions of the equipment were as follows: the RF power was set at 1500 W, the plasma gas flow at 10 L/min, the auxiliary gas flow at 2.0 L/min, the nebulizer flow at 0.7 L/min and the pump flow rate at 1 mL/min. Systems comprising H.p. Mg, PEO, and PEO/PCL (comprising 15 mg) in the absence or presence of the drug, with a surface area of approximately 12 cm^2^, were immersed in 25 mL of inorganic α-MEM solution at 37 °C. At intervals of 1 h and 24 h, a 2 mL aliquot was extracted from the testing solution, and subsequently, fresh medium was introduced to the solution in order to maintain constant volume. The blank of inorganic α-MEM was incorporated into the batch measurements as a benchmark. It is important to note that each measurement was conducted in triplicate, with a relative standard deviation (RSD) of less than 3%.

## 3. Results

### 3.1. Characterization of the PEO- and PEO/PCL-Treated Surface

#### 3.1.1. Optimization of PEO Coating

**Figure 1:**
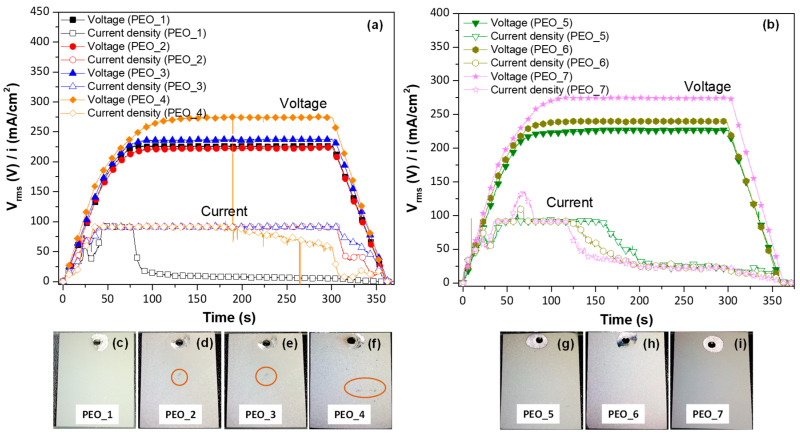
Current density-time curves for PEO coatings at different frequencies: (**a**) 50 Hz and (**b**) 400 Hz. (**c**–**i**) Digital images of PEO coatings formed. Size of the sample 3 cm × 2.3 cm. The orange circles correspond to the defects formed during the PEO process.

The evaluated PEO parameters are illustrated in Table 2, the voltage range and frequency suggested by a previous study [35]. Figure 1 shows the voltage and current density curves acquired during the PEO process at two different frequencies: (a) 50 Hz and (b) 400 Hz, with the increase in the input voltage from 360 V to 450 V. The digital macrographs corresponding to these PEO coatings are displayed in Figure 1c–i.

In Figure 1, all PEO curves show a linear increase in voltage up to 60 s, with a change in slope around 40 s (160 V), which is due to the current density having achieved the limit at 100 mA/cm^2^. The second inflexion point corresponds to the dielectric breakdown of the material at ~210–220 V, where plasmochemical reactions lead to the formation of the coating. Microdischarges formed on the surface of all samples, but they were not visible due to the electrolyte’s opacity from suspended CaO. At 80 s, a constant voltage of 230–270 V_rms_, depending on the applied waveform amplitude, is reached and maintained until the end of the PEO process. It is worth mentioning that in the case of PEO_1, PEO_4, PEO_5, PEO_6, and PEO_7, a drop in current density is observed. This phenomenon indicates an improved coating impedance and a change in coating growth due to an increase in the resistance of the oxide material to mass and charge transfer [36]. After the drop, the current density continued its gradual decrease until the process was stopped (final 60 s ramp). In the case of PEO_1, no characteristic sound of plasma microdischarges could be heard from 100 s on (i.e., at current density ≤ 5 mA/cm^2^), suggesting that the process was continued as conventional anodizing, which does not lead to a coating thickness increase but repairs the internal micro-defects [37].

The digital images of the coatings formed at low frequency (50 Hz) show a more uniform surface for PEO_1 (Figure 1c), while surface irregularities are observed in the coatings formed at higher voltages (Figure 1d–f, orange circles). In comparison, the appearance of the coatings formed at 400 Hz is overall more uniform (Figure 1g–i) due to the fact that the micro-cracks are minimized, which is in concordance with other works [38,39].

The increase in voltage amplitude at both frequencies (50 and 400 Hz) leads to an increase in thickness from 5 to 8.7 µm and from 5 to 6.4 μm, respectively, being higher at 50 Hz (Table 2). The latter is consistent with a higher charge passed during the positive pulse [40,41]. However, the highest positive pulse (400 V, PEO_4) in combination with low frequency leads to a slight reduction in thickness (from 8.7 to ~8 µm), which can be attributed to longer-lived microdischarges that promote partial destruction of the newly formed oxide material [42]. The increase in the frequency from 50 to 400 Hz leads to a thickness loss (from ~8 to ~5 μm), which is consistent with the early current drop (hence the lower charge) in the 400 Hz specimens, compared with their 50 Hz counterparts.

The apparent specific energy consumption calculated for the PEO-coated samples is illustrated in Table 2. It is evident that the energy consumption values are mostly affected by the current drop time, the lowest energy of 3.7 kW·h/m^2·^µm^−1^ corresponding to the PEO_1 treatment. The shortest current drop time in this case can be attributed to the lower amplitude of the cathodic pulse (−30 V). For comparison, when cathodic amplitude of −50 V is used (PEO_2), no current drop occurs, and the energy consumption nearly triples as a result (9.5 kW·h/m^2·^µm^−1^). Nominé et al. have pointed out that microdischarges can take place during the cathodic pulse [43]. Their intensity (i.e., light emission) can be even more intense than from the anodic microdischarges, if cathodic bias is large enough. These cathodic microdischarges cause spalling of the oxide material [44], which explains why the coating impedance is low, hence no current drop and high energy consumption. An increase in the operating frequency induces a faster interruption of anodic microdischarge, minimizing its destructive effect, hence greater coating impedance and earlier current drop.

#### 3.1.2. Selection of the PEO Coating by EIS

**Figure 2:**
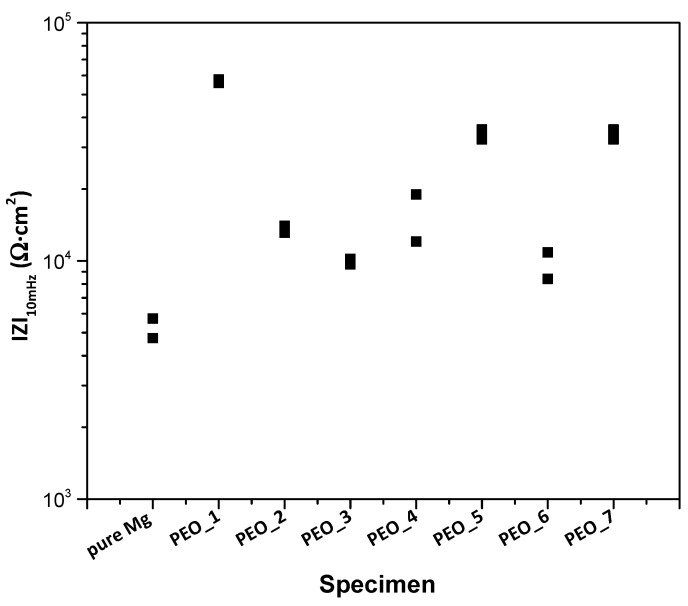
Scatter diagram of the impedance values for the studied PEO coatings after 1 h of immersion in modified α-MEM. Duplicate experimental data points are shown in the graph.

In order to select the best PEO coating, a screening of the PEO coatings after 1 h immersion in a modified α-MEM solution is carried out. Figure 2 shows the total modulus of impedance at low frequencies (|Z|_10 mHz_) for all PEO coatings.

In general terms, all coatings exhibit an increase in corrosion resistance compared to the bulk material. It should be noted that for the group of coatings formed at low frequency (50 Hz), |Z|_10 mHz_ is the highest for the lowest peak-to-peak voltage (PEO_1, 360 V U_p-p_), and its corrosion resistance does not increase despite the increase in coating thickness. There is no clear trend in corrosion behaviour of the group of coatings formed at 400 Hz, and, overall, its corrosion resistance is lower than that of PEO_1. Comparing both frequencies for the same peak-to-peak voltage, increasing the frequency leads to an increase in corrosion resistance, which is in agreement with the literature [45]. However, similar corrosion resistance is observed for PEO_3 and PEO_6 (400 V U_p-p_), which could be associated with the morphology and porosity of the coatings.

It is worth mentioning that the PEO_1 specimen with the lowest frequency, voltage and thickness presents the best corrosion behaviour compared to the other coatings. This could be due to (i) the employment of softer electrical parameters that avoid the generation of more aggressive sparks that could produce an undesired high porosity; it could also be due to (ii) the early current drop that enabled the repairing of internal nano-defects in the coating by conventional oxidation at 5–10 mA/cm^2^. This has resulted in a more compact and corrosion-resistant coating, which is in accordance with the observed reduction in current density (Figure 1a). Therefore, the PEO_1 coating (further referred to as PEO) is selected for detailed characterization and for the formation of a hybrid coating due to its better corrosion behaviour.

#### 3.1.3. Characterization of PEO and Hybrid PEO/PCL Coatings

**Figure 3:**
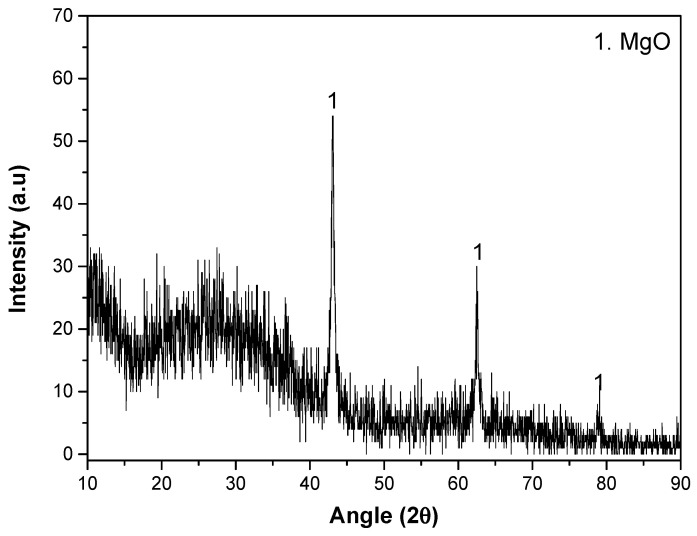
XRD pattern of PEO system.

The XRD pattern of the coating is displayed in Figure 3. The coating reveals MgO as the only crystalline phase. In addition, the presence of amorphous material corresponds to a peak broadening between 15° and 40° (2θ). A certain degree of background noise and signal broadening can also be observed, which is characteristic of PEO coatings. This effect is due to the high surface roughness, heterogeneous thickness and porosity of the oxide layer, as well as the coexistence of crystalline and amorphous domains generated during rapid solidification in micro-discharges. The lack of apatite or hydroxyapatite is understandable, as a higher positive pulse voltage (i.e., higher plasma discharge temperature and lower cooling rate of the material) is normally required for its formation [46].

**Figure 4:**
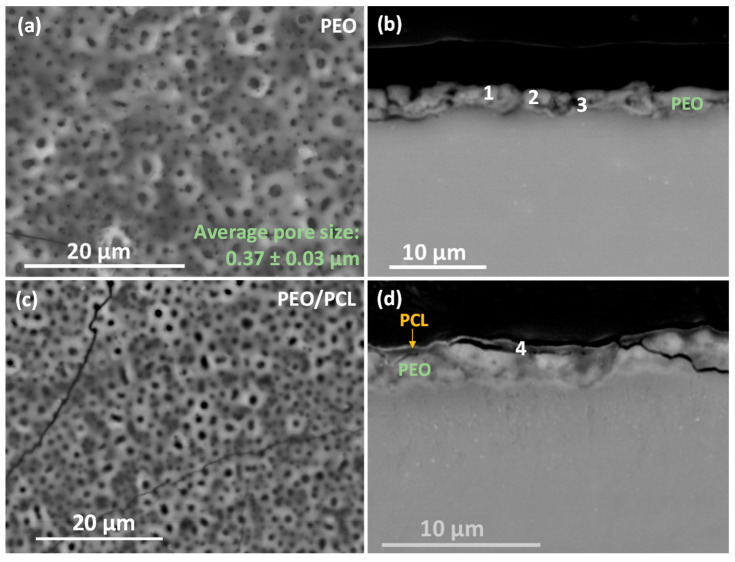
(**a**,**c**) Secondary electron micrographs of surface morphology and (**b**,**d**) backscattered electron micrographs of cross-sections of (**a**,**b**) PEO and (**c**,**d**) PEO/PCL specimens. EDS analysis locations correspond to the data in Table 3.

Figure 4 shows the top views and cross-sectional images of (a,b) PEO and (c,d) duplex PEO/PCL coatings, and their EDS analyses are displayed in Table 3. The surface of the PEO coating shows a typical microporous morphology with an average pore size of ~0.4 µm (Figure 4a). These regularly distributed micropores are the result of discrete localized discharge events associated with dielectric breakdown and the expulsion of the generated gas (O_2_ and H_2_) through the molten metal oxide. Considerable amounts of P, Si and Na elements detected by the EDS analysis (PEO plan view, Table 3) are derived from the electrolyte. In the case of the PEO/PCL specimen (Figure 4c), the surface EDS analysis detected a large amount of C corresponding to the presence of PCL. However, the pores are still visible, as the PCL layer is ~0.3 μm-thick (Figure 4d).

The cross-sectional images (Figure 4b,d) show a uniform morphology of the ceramic PEO layer with a thickness of around 2.5 μm and small internal pores in the coating. Figure 4d discloses a sub-micron-thick barrier layer. The incorporation of P, Si and Na decreases towards the substrate (Table 3, points 1–3). It is worth mentioning that the incorporation of Ca in the coating is minimal (hence the low Ca/P ratio), which may be due to a reduction in the mass transfer of ions from the electrolyte towards the substrate due to the decrease in the current density observed in Figure 1a. The PCL layer is composed of C and O (Table 3, point 4), the main composition of the polymer structure.

#### 3.1.4. Corrosion Behaviour in H_2_ Evolution Test

**Figure 5:**
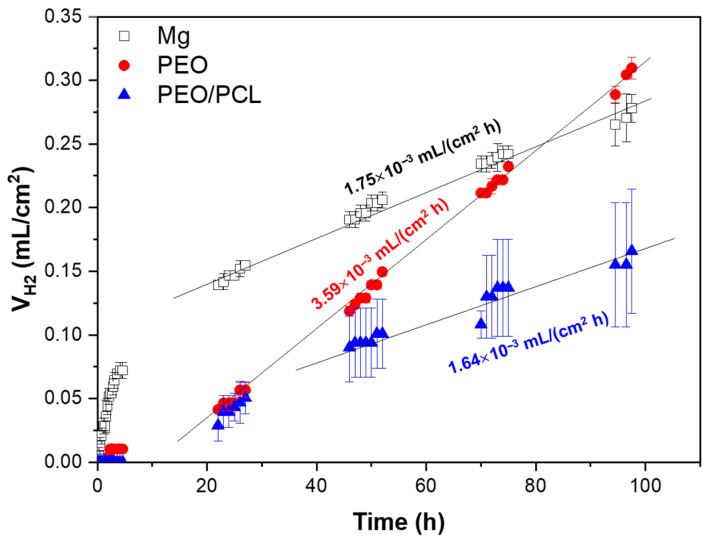
Hydrogen evolution test of Mg, PEO and PEO/PCL after 4 days of immersion in inorganic α-MEM solution, controlling the pH at 7.4 by CO_2_ flow. The straight lines in the graph correspond to the linear trend of corrosion in the steady state set.

The hydrogen evolution test was performed to evaluate the corrosion behaviour of the substrate, PEO- and PEO/PCL-coated samples during four days of immersion in modified α-MEM, controlling the pH constant at 7.4 by CO_2_ flow (Figure 5).

**Figure 6:**
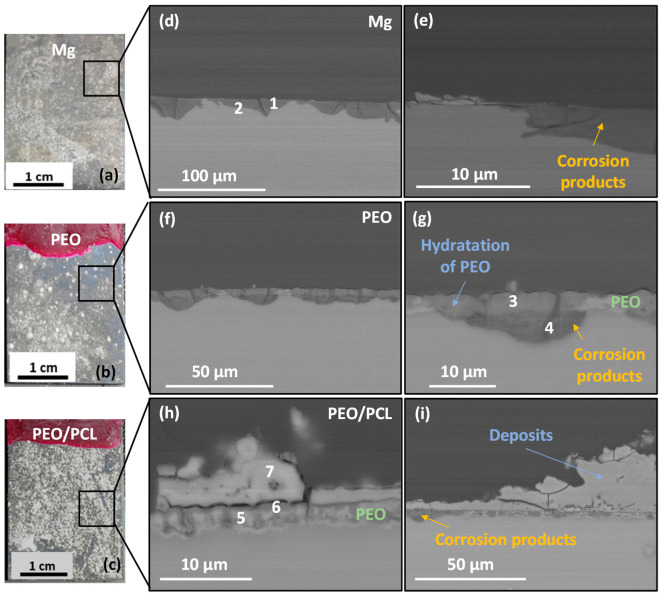
(**a**–**c**) Photos and (**d**–**i**) backscattered electron micrographs of cross-sections of (**d**,**e**) h.p. Mg, (**f**,**g**) PEO and (**h**,**i**) PEO/PCL after 4 days of immersion in modified α-MEM solution at 37 °C. EDS analysis locations correspond to the data in Table 4.

The h.p. Mg specimen (with 45.5 ppm Fe, Table 1) shows a high hydrogen release rate during the first hours of immersion, which slows down and stabilizes after 24 h of immersion at 1.75 × 10^−3^ mL/(cm^2^·h), which is equivalent to ~0.09 mm/year. For comparison, Mei et al. observed a ~1.27 mm/year corrosion rate in TRIS-free SBF for commercially pure Mg that contained 342 ppm of Fe [47]. It has been shown previously that Fe impurity content is the determining factor in the corrosion resistance of pure and ultra-pure Mg alloys [10,11]. In the case of the PEO- and PEO/PCL-coated specimens, a stabilization of the corrosion rate occurs after ~24 h and ~48 h of immersion, respectively. Both coated systems show about one order of magnitude reduction in H_2_ evolution volume within the first 24 h compared with the substrate, which is associated with the protective capacity of the ceramic and hybrid layers [48]. However, after 24 h, the PEO-coated system shows a remarkable two-times-higher corrosion rate than the bare substrate (3.59 × 10^−3^ mL/(cm^2^·h) or ~0.18 mm/year), so that its actual Mg loss outstrips that of the substrate by 96 h of immersion. This acceleration can be attributed to the steady hydration of the PEO coating that facilitates the ingress of Cl^−^ ions into the crevice that forms under the coating. The crevice-induced corrosion issues of Mg alloys have been observed before by Wu [49] and Moreno [31], where the crevice microenvironment becomes more concentrated in Cl^−^ and therefore more aggressive. This is corroborated by the greater amount of Cl (0.6 at.%) detected by EDS in the undercoating corrosion products layer as compared to 0.1–0.2 at.% found in the corrosion layer on PEO-free substrate, as seen (Figure 6d,g, Table 4). The PEO/PCL system, on the other hand, showed zero H_2_ evolution in the first 5 h, due to the sealing PCL layer effectively impeding the initial penetration of the corrosive species towards the substrate. The corrosion rate of the PEO/PCL system between 48 h and 96 h is equivalent to ~0.08 mm/year.

According to the literature, the corrosion current density of a ~40 μm thick hybrid PDA-PTMC/PEO coating on 99.98% pure Mg in PBS was about three times lower than that of a stand-alone PEO system, which was attributed to the sealing of the pores and cracks of the PEO coatings by the polymeric (PDA and PTMC) coatings [18]. A similar situation has been observed in the present work: ~2.5 μm thick PEO/PCL coating showed a two times lower volume of accumulated H_2_ during 48–96 h immersion period compared with bare h.p. Mg substrate.

Figure 6 shows the macrographs and cross-sectional images of the three studied samples after four days of immersion in modified α-MEM. The macrographs of the samples (Figure 6a–c) show a silver-grey patina and white deposits on the surface, which correspond to corrosion products. In the case of h.p. Mg, a 5–15 μm thick corrosion product layer is observed (Figure 6d,e). For the PEO- and PEO/PCL-coated systems, corrosion develops uniformly under the progressively hydrated coating, which remains adhered to the sample (Figure 6f–i). The corrosion products formed in all cases present high P, Si and Ca content (Table 4, points 1–3), which is typical of Mg passivation in body fluids [50].

It is worth mentioning that in the case of PEO/PCL specimen (Figure 6c,h,i) and Supplementary Figure S1, the PCL layer does not appear to be present anymore, suggesting that it may have been detached, possibly within first 24 h of immersion, which correlates with the fact that the hydrogen volume released between 24 and 30 h of immersion catches up with that for PEO-coated system (see Figure 5). Furthermore, a 5–40 μm thick and compact layer of C, Ca- and P-rich products has precipitated on the surface of the PEO/PCL sample (Table 4, point 7). The precipitation of the species such as Ca(CO_3_) and (Ca_5_PO_4_)_3_(OH) from the physiological medium is facilitated by the hydrolysis of silica in the PEO coating, as discussed in our previous work [51]. The alkalization of the modified α-MEM solution needed for such precipitates is pH > 7.0. Considering that the continuous CO_2_ injection adjusts the bulk pH to 7.4, it appears that the crevice generated between the PEO and PCL layers during the corrosion process [24] helps to maintain the near-surface alkalinization at the level needed to trigger the precipitation. In bone regeneration applications, the capacity of PEO/PCL hybrid systems to induce such precipitates is expected to assist bone remineralization. These precipitates also visibly impede the hydration of the PEO layer, help reduce the extent of the undercoating corrosion (Figure 6h,i) and preserve the barrier layer mostly intact. In other words, the Ca-P deposit prevents the formation of the undercoating crevice rich in Cl^−^, which explains the lower volume of hydrogen released after ~40 h compared to the PEO sample that lets the Cl^−^ filter through and accumulate in the underlying corrosion layer (Figure 5). Notably, no pitting was found in the entire 23 mm long cross-section of the embedded PEO/PCL specimen.

**Figure 7:**
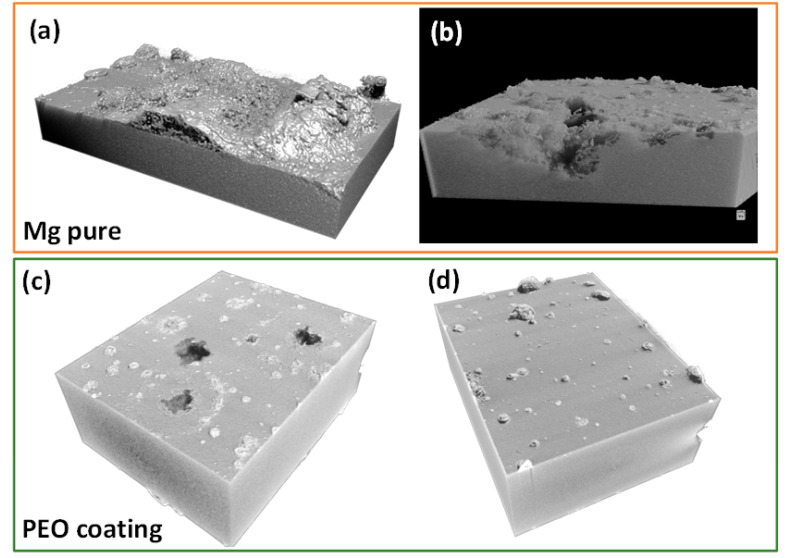
Micro-CT 3D images of specimens after 4 days of immersion: (**a**,**b**) h.p. Mg and (**c**,**d**) PEO coatings at different regions of the sample.

Micro-CT was used to assess the extent of the localized corrosion propagation in bare and PEO-coated substrates after four days of immersion in modified α-MEM. The corrosion of bare Mg is much more severe than that of the PEO-coated specimen, with a penetration depth of corrosion damage ~1 mm at some places, as shown in Figure 7a,b. However, in the case of the PEO coating, there is mostly uniform corrosion, a few pits and regions covered with deposits, possibly associated with precipitation of Ca, P-rich products from the media, as it was discussed before. The videos provided in Supplementary Figure S2 disclose that the most prominent pits are ~0.5 mm deep and ~0.3 mm wide.

### 3.2. Screening of the Pharmaceutical Agents

#### 3.2.1. Electrochemical Impedance Spectroscopy (EIS)

In order to select a drug for surface functionalization, the corrosion modulating effect on h.p. Mg was evaluated using the following pharmaceutical agents: (i) anti-inflammatory agents: aspirin, paracetamol and naproxen; and (ii) antibiotics: gentamicin, ciprofloxacin and streptomycin sulphate. These agents are frequently administered orally or intravenously as part of a post-surgical treatment, i.e., they are constantly present in the bloodstream during 5–10 days after surgery. Antibiotics are of particular interest, as they can be incorporated into the implant surface and provide a local antimicrobial effect to combat common post-operative infections. If these agents act as corrosion inhibitors, they have added value, as they help to control the implant degradation rate. However, it is even more important to know whether a clinically prescribed drug is a corrosion accelerator, because in that case, the coating system must ensure extra protection. Our goal here is to identify agents with the worst possible effect on the corrosion rate of h.p. Mg.

**Figure 8:**
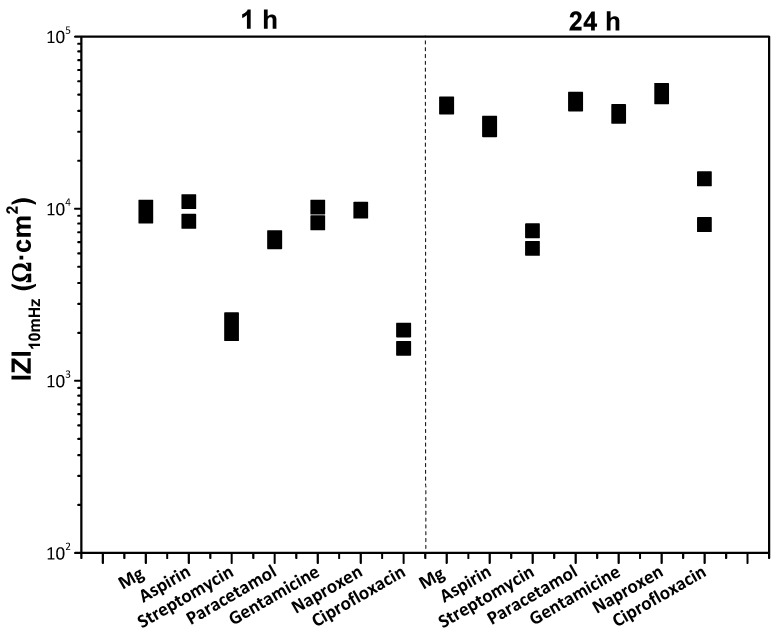
Scatter diagram of the impedance values of bare h.p. Mg substrate in the presence of different pharmaceutical agents after 1 and 24 h of immersion. Duplicate experimental data points are shown in the graph.

Figure 8 shows the total modulus of impedance at low frequencies |Z|_10 mHz_ for the bare Mg substrate after 1 h and 24 h of immersion in modified α-MEM solution. It discloses that streptomycin, paracetamol and ciprofloxacin, with lower |Z|_10 mHz_ after 1 h of immersion compared to the bulk material, act as corrosion accelerators. The rest of the pharmaceutical agents show a similar impedance to that of the substrate in the drug-free medium, which suggests that they are not modulating the corrosion of Mg. After 24 h, streptomycin and ciprofloxacin still show an accelerating effect on magnesium corrosion, while paracetamol shows the same corrosion behaviour as the substrate without drugs. The latter effect may be attributed to the overall increase in |Z|_10 mHz_ with time, which is due to the formation of a corrosion product layer that in itself has some protective properties [50]. Given that a rapid (24 h) immersion test may possibly not have been discriminating enough, it was decided to evaluate all pharmaceutical agents by hydrogen evolution measurements for 96 h, a period comparable with the duration of a standard in vitro cell proliferation assay. This was performed to verify the results of the EIS screening and further discriminate the pharmaceutical agents.

#### 3.2.2. Corrosion Behaviour by H_2_ Evolution Test

**Figure 9:**
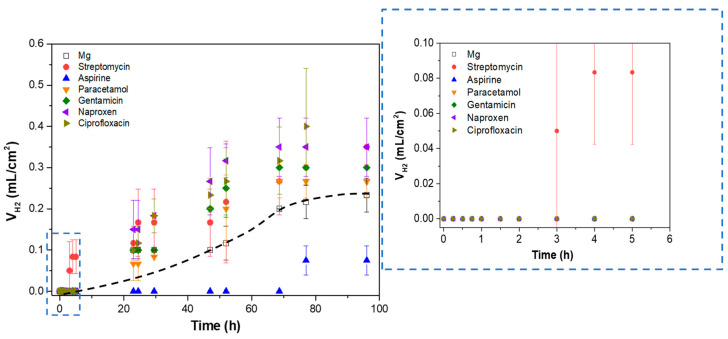
Hydrogen evolution test of h.p. Mg exposed to different pharmaceutical agents after 96 h of immersion in inorganic α-MEM solution. The dotted black line represents the corrosion rate trend in the h.p magnesium sample.

Figure 9 shows the volume of hydrogen released after 96 h of immersion of h.p. Mg in a modified α-MEM with 120 µg/mL of added drugs.

In general, all the agents except streptomycin show no perceptible hydrogen release during the first 5 h of immersion, which is in accordance with the results observed in EIS after 24 h of immersion (Figure 8). After 24 h of immersion, a linear increase in released hydrogen volume is observed, followed by a stabilization of the curve after 70 h and until the end of the test, which may be associated with the formation of a stable corrosion product layer that protects the material against corrosion [52]. Interestingly, aspirin is the only agent that at all times presents at least a ~two times lower hydrogen release compared to the substrate in the drug-free medium. This phenomenon may be associated with the formation of ASP-Me(II) complexes (Me(II) being Ca or Mg cations) [53] on the surface of the samples, blocking the access of corrosive species and reducing the corrosion rate, similarly to the effect of aspirin on the corrosion of stainless steel [54].

Taking into account the overlap in standard deviation values during the hydrogen test, the differences between the corrosion-accelerating agents cannot be considered statistically significant. Of all these pharmaceutical agents, streptomycin was the only drug that induced a higher corrosion rate during the first 8 h of immersion. Therefore, it was selected for surface functionalization of the PEO/PCL system, as potentially the most corrosive agent, in order to test the protective capacity of the hybrid coating. While several NSAIDs demonstrated corrosion-accelerating behaviour in solution, these were not selected for incorporation into the hybrid system. The objective was not to optimize the coating for a specific therapeutic application, but rather to challenge its performance under the most aggressive conditions. The preliminary screening identified streptomycin as the most corrosive drug, thus establishing it as the most suitable candidate for probing the system’s protective limits. Furthermore, earlier research has demonstrated that, despite their corrosive properties, non-steroidal anti-inflammatory drugs (NSAIDs), including naproxen and paracetamol, do not result in the passivation of Mg surfaces or the formation of protective complexes [55,56].

#### 3.2.3. Surface Characteristics of Functionalized Duplex System

**Figure 10:**
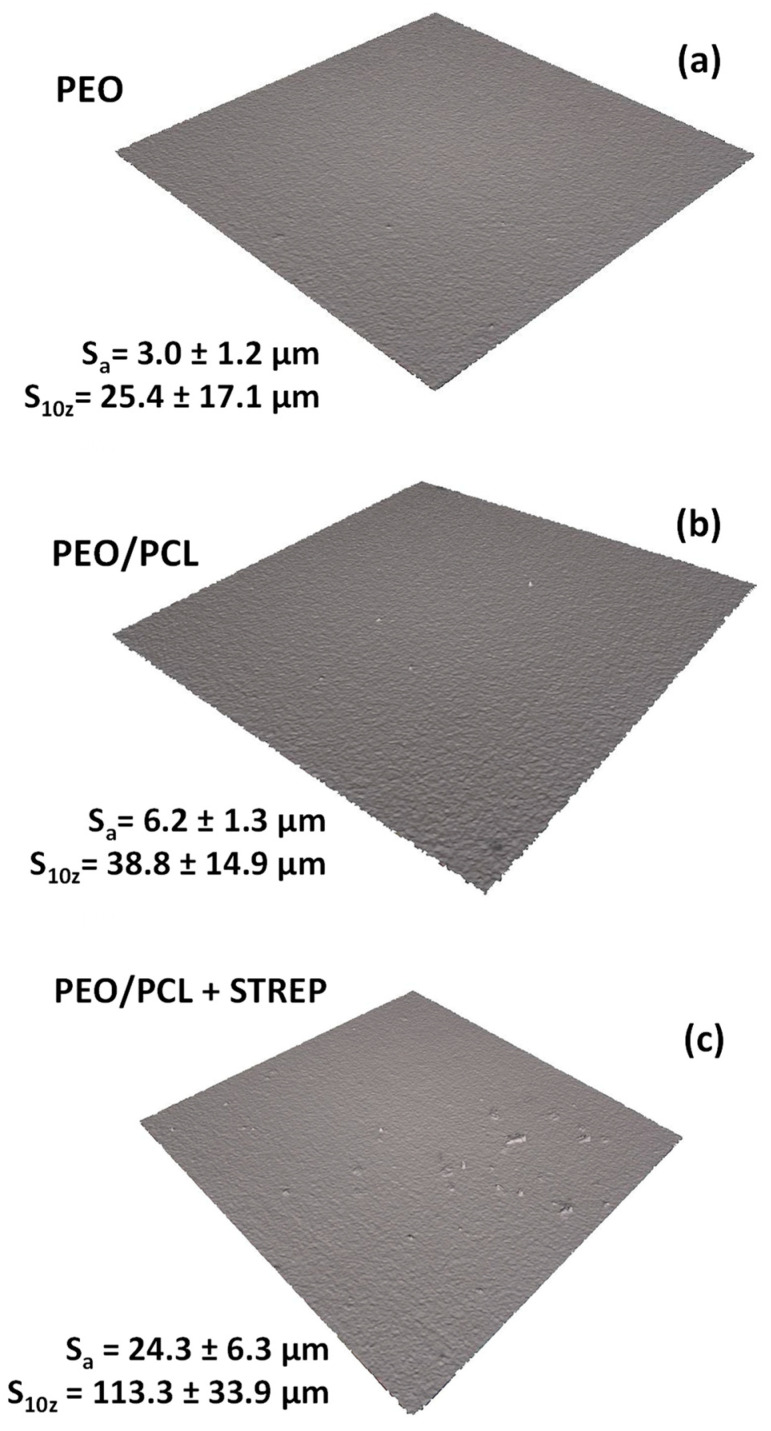
Optical profilometry micrographs of PEO, PEO/PCL and PEO/PCL with streptomycin (STREP) systems (**a**–**c**) using 3D rendering of the surface.

**Figure 11:**
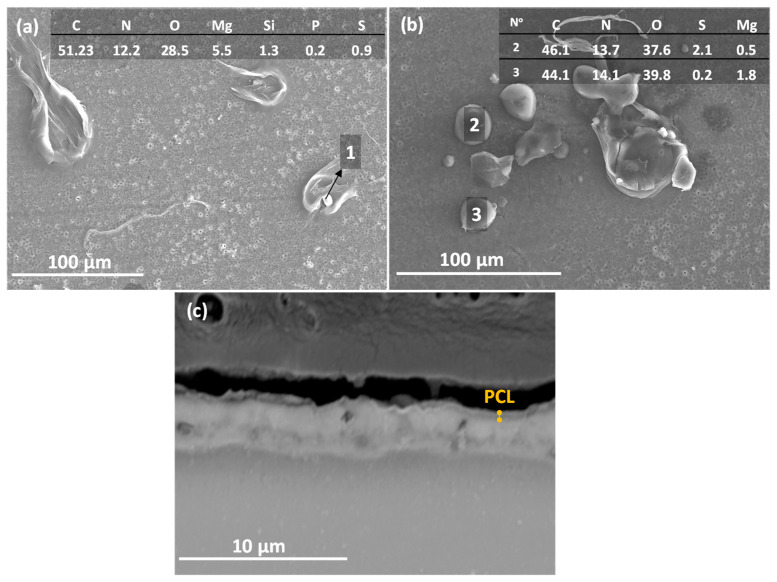
(**a**,**b**) Top view and EDS analysis at% and (**c**) cross-section SEM images of the PEO/PCL + Streptomycin. The numbers of (**a**,**b**) correspond with the EDS analysis at% of the granules.

Figure 10 illustrates the 3D optical micrographs for (a) PEO, (b) PEO/PCL and (c) PEO/PCL + Streptomycin specimens. The surface of the PEO/PCL system was found to be rougher compared to PEO (S_a_ increased from ~3.0 to ~6.0 μm), which could be attributed to the thickening effect of the polymeric layer applied over the porous and rough PEO layer, and has also been observed in other works [19,57]. The incorporation of streptomycin increases the roughness by four times because the drug is incorporated into the PCL layer in the form of granules (Figure 11a,b). In Figure 11a, some deposits trapped underneath the PCL polymer layer can be seen, whose main components are C, N, S and O, the elements of the streptomycin structure, confirming its incorporation into the coating. It should be noted that streptomycin solubility in chloroform is negligible, i.e., the powder is simply dispersed in PCL/chloroform solution, hence the different size of the granules. There are regions (Figure 11b) where some granules have not been properly incorporated, and deflated air bubbles have formed. This may be associated with their large size, which causes the polymer coating to stretch and break [58], leading to the loss of the deposits. Cross-sectional examination (Figure 11c) did not disclose any streptomycin granules under the ~0.5 µm thick PCL layer, but that may be related to this specific region of examination.

**Figure 12:**
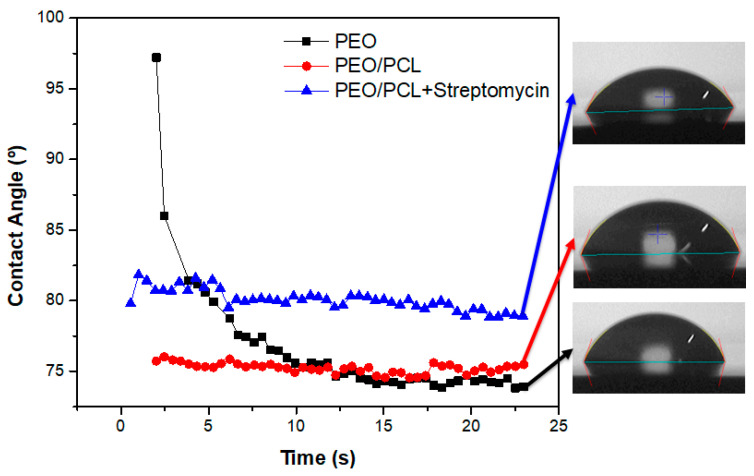
Evolution of water contact angle of coated systems during 25 s. The black-square correspond to PEO sample, the red-circle to the PEO/PCL sample and the blue-triangle to the PEO/PCL+Streptomycin sample. The drops images correspond to the last image taken at 22 s according to the studied sample.

The wettability of the PEO, PEO/PCL and PEO/PCL + Streptomycin systems has also been evaluated because of its great importance to cell adhesion. The evolution of the water contact angle with time for these specimens is illustrated in Figure 12. The contact angle for the PEO coating drops fast over time from 98° to 73°, which may be due to the porous surface of the coating, where liquid is drawn into the pores of the coating. However, in the case of the duplex system with and without streptomycin, the contact angle remains constant over time at 75° and 80°, respectively, being higher in the case of the PEO/PCL + Streptomycin system. This phenomenon may be attributed to the hydrophobicity of the streptomycin sulphate compound [59].

#### 3.2.4. Corrosion Behaviour of Functionalized Hybrid System

**Figure 13:**
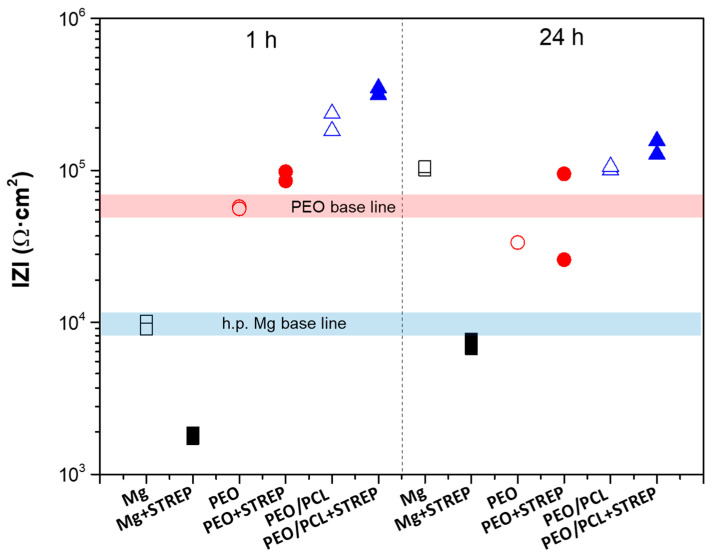
Scatter diagram of the impedance values for h.p. Mg (black squares), PEO (red circles) and PEO/PCL (blue triangles) with (filled symbols) and without (open symbols) streptomycin (STREP) after 1 and 24 h of immersion. Duplicate experimental data points are shown in this graph.

In order to assess the effect of streptomycin on degradation behaviour, the |Z|_10 mHz_ values of h.p. Mg, PEO and PEO/PCL systems with and without streptomycin have been obtained after 1 h and 24 h of immersion in modified α-MEM at 37 °C (Figure 13). Their comparison reveals an important phenomenon: while streptomycin accelerates the corrosion of bare h.p Mg, it acts as a corrosion inhibitor in the presence of PEO and PEO/PCL coatings. This becomes particularly evident after 24 h of immersion, when the|Z|_10 mHz_ value of PEO in a drug-free medium drops below its 1 h baseline, but PEO + Streptomycin and PEO/PCL + Streptomycin remain on par and above it, respectively. Also, the streptomycin-functionalized PEO/PCL system performs better than its drug-free counterpart both after 1 h and 24 h of immersion.

**Figure 14:**
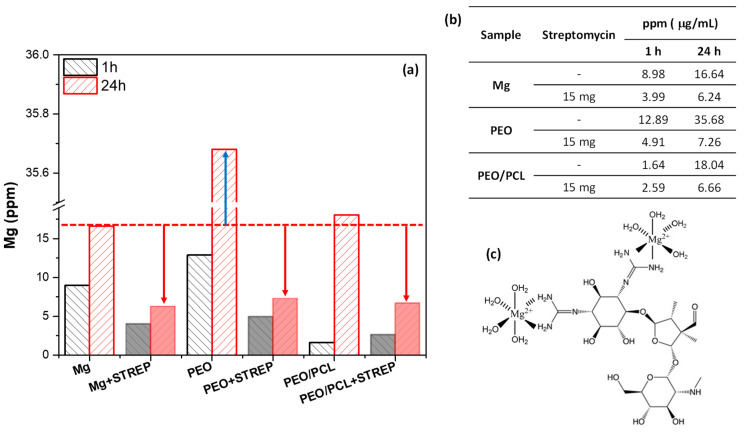
(**a**) Mg ion release from h.p. Mg, PEO and PEO/PCL with and without streptomycin (STREP) after 1 and 24 h of immersion in modified α-MEM solution. (**b**) Precise values from (**a**). (**c**) Structure of the insoluble Me(II)-Streptomycin chelates, where Me (II) correspond to Mg^2+^ and Ca^2+^ ions. The arrows in the (**a**) correspond to the distance between the value of ppm of the substrate without streptomycin and the h.p Mg, PEO, PEO/PCL samples of study with streptomycin.

This inhibiting effect could be associated with the formation of insoluble chelates such as Streptomycin–(Mg/Ca), which block the PEO pores and the access of corrosive species, reducing the degradation rate of both systems. In order to verify the interaction of streptomycin with Mg^2+^, the Mg ion release from h.p. Mg, PEO and PEO/PCL systems was measured over 24 h in the modified α-MEM with and without the presence of streptomycin (Figure 14). Three trends were observed after 24 h of immersion: (i) All samples with and without streptomycin exhibit higher Mg release than at 1 h of immersion, suggesting that the corrosion is in progress for all study systems. (ii) The PEO system without streptomycin shows higher Mg release by ~two times than the bare substrate and the PEO/PCL system. This phenomenon may be associated with the lixiviation of Mg^2+^ from oxide material and corrosion products, due to the high permeability of the coating, hydration of MgO and solubilization of the resultant Mg(OH)_2_ (K_sp_ Mg(OH)_2_ = 5.61 × 10^−12^), solubility 12.2 mg/L [60,61]. (iii) All samples with streptomycin exhibit lower Mg release by 2–3 times after 24 h immersion.

The low Mg release from the specimens with streptomycin may be associated with the formation of insoluble Me(II)-Streptomycin chelates, due to the interaction of the streptomycin released from the coatings with Mg^2+^ and Ca^2+^ ions. The latter are easily lixiviated from the PEO layer. The precipitate formed in the solution would not be detected in the aliquot taken for the ICP-OES. A report has demonstrated that streptomycin can form chelating complexes with some metal ions, such as silver, copper, nickel or cobalt [62,63], so it could form complexes with calcium (Ca) and magnesium (Mg), consistent with what has been observed here. It is well known that these cations limit the uptake of streptomycin [64]. This evidence corroborates the obtained electrochemical results for the drug-functionalized systems, where these chelates would precipitate in the pores of the PEO coating and become trapped there within the hydrolyzed silica. The blocked passage of corrosive species through the cracks and pores of the coating system explains the increase in corrosion resistance of PEO + Streptomycin and PEO/PCL + Streptomycin systems (Figure 13). Similar conclusions were reached in our previous work regarding the inhibitory effect of ciprofloxacin loaded in a hybrid hierarchical coating system constituted by porous polymer and PEO coating on a Mg-Zn-Ca alloy [31].

In the case of bare h.p. Mg, the ICP-OES results (~4 ppm of detected Mg in the presence of streptomycin vs. ~9 ppm without, Figure 13) at first appear to contradict the low |Z|_10 mHz_ values for Mg-Streptomycin (Figure 13). However, it must be remembered that the ICP-OES measurements were performed in 2 mL aliquots taken from the medium, and therefore, Mg^2+^ ions bound into Me(II)-Streptomycin chelates (as loose precipitates in the solution) were not detected. The Mg^2+^ cations released as a result of the corrosion process and captured by streptomycin change the equilibrium so that there is a stronger shift to the right in the reaction Mg + 2H_2_O ⟶ Mg(OH)_2_ + H_2_, leading to the acceleration of the corrosion process. For this reason, the corrosion resistance of the Mg–Streptomycin system is lower compared to loaded-coated systems.

In summary, the corrosion-modulating effect of streptomycin on h.p. Mg depends on the studied system, exhibiting an inhibitory effect in the presence of a porous ceramic or hybrid coating, while accelerating the corrosion of the bare substrate. This appears to be associated with the formation of insoluble Streptomycin–(Ca/Mg) chelates that clog the PEO pores and prevent the ingress of corrosive species towards the barrier layer of PEO coating. In the case of bare h.p. Mg, the chelate complexes do not have the same “repairing” effect on Mg(OH)_2_ layer. In the case of a hybrid PEO/PCL coating system, the C, Ca- and P-rich deposits induced by the pH alteration in the PEO/PCL crevice afford further protection.

## 4. Conclusions

- A range of AC PEO conditions developed for h.p. Mg in an electrolyte containing bioactive species was evaluated with respect to coating thickness and corrosion resistance, varying voltage amplitudes and frequency. The increase in peak-to-peak voltage led to an increase in the coating thickness in the range of 6–8 µm, while the increase in frequency led to a decrease in thickness from 8 to 5 µm. However, milder sparking conditions (the lowest peak-to-peak voltage) resulting in early current drop and enhanced compactness of the coating were found to be the determining factor in the corrosion resistance of a stand-alone PEO coating constituted by MgO and amorphous phase.
- A hybrid PEO/PCL coating on h.p. Mg prevented the onset of corrosion during at least 5 h, reducing the amount of evolved hydrogen gas during the following period by two times, equivalent to a degradation rate of 80 μm/year. This was due to the formation of Ca-P compounds on the surface, blocking the access of corrosive species. The precipitation is thought to be facilitated by local alkalinization in the crevice between the PEO and PCL layers.
- For screening of the corrosion-modulating effect of pharmaceutical agents on h.p. Mg, a 96 h long hydrogen evolution test was found more reliable and discriminating than a 24 h long EIS screening. Gentamicin, naproxen, streptomycin, ciprofloxacin and paracetamol (in no particular order) were found to be corrosion accelerators for bare h.p. Mg, whereas aspirin was found to inhibit the corrosion by ~two times. Streptomycin exhibited the accelerating effect sooner than other drugs, within 8 h of immersion.
- Streptomycin inhibited corrosion of both stand-alone PEO and hybrid PEO/PCL coating systems on h.p. Mg. This effect can be considered an active corrosion protection mechanism, as it is triggered upon the release of the coating and substrate cations, followed by the coating defect-blocking action of the precipitated insoluble chelates of Mg^2+^ and Ca^2+^ cations with streptomycin.

## Figures and Tables

**Table 1:** Composition in at.% of Mg.

Mg	Ag	Al	Ca	Ce	Cu	Fe	La
99.915	0.000075	0.01235	0.005925	0.001865	0.00092	0.00455	0.00065
Mn	Ni	Pb	Si	Sn	Zn	Zr	Be
0.02895	0.000725	0.000205	0.0265	0.0004	0.002195	0.00275	0.000021

**Table 2:** Process parameters employed for the selection of PEO coating.

Sample	V_p-p_ (V)	Frequency (Hz)	Thickness (μm)	E_app_ (KW·h/m^2^·µm)
**PEO_1**	360	50	3.0 ± 0.6	3.70
**PEO_2**	380		5.0 ± 0.5	9.53
**PEO_3**	400		8.7 ± 0.5	5.96
**PEO_4**	450		8.0 ± 1.0	6.31
**PEO_5**	380	400	5.0 ± 0.4	6.26
**PEO_6**	400		5.4 ± 0.3	5.48
**PEO_7**	450		6.4 ± 0.4	4.93

**Table 3:** EDS analysis (at.%) of PEO and PEO/PCL specimens.

Sample	Point	C	O	Na	Mg	Si	P	K	Ca	Ca/P
**PEO**	Plan view	23.0	47.5	0.4	22.6	4.6	1.8	0.02	0.04	0.02
	1	55.5	30.7	0.2	10.5	2.4	0.7	0.01	0.01	0.02
	2	40.9	35.2	0.2	19.6	3.3	0.9	0.00	0.02	0.02
	3	21.3	27.5	0.1	46.3	4.2	0.6	0.01	0.01	0.02
**PEO/PCL**	Plan view	67.0	20.6	0.1	9.7	2.5	-	0.01	-	-
	4	82.0	15.7	0.01	2.0	0.5	-	0.01	-	-

**Table 4:** EDS analysis (at.%) of Mg, PEO and PEO/PCL after 4 days of immersion.

Sample	Point	C	O	Na	Mg	Si	P	Cl	Ca	Ca/P
**h.p. Mg**	1	31.2	54.5	0.0	13.5	0.0	0.5	0.2	0.1	6.71
	2	33.4	47.4	0.4	5.4	0.1	6.3	0.1	7.0	0.91
**PEO**	3	46.2	40.4	0.1	7.2	3.8	1.7	0.1	0.6	0.35
	4	38.5	44.7	0.02	15.4	0.4	0.3	0.6	0.1	0.40
**PEO/PCL**	5	21.0	52.3	0.3	19.9	4.4	1.6	0.01	0.6	2.73
	6	26.9	52.9	0.3	2.6	0.1	8.3	-	9.1	0.91
	7	24.3	54.3	0.3	10.0	3.0	4.3	0.02	3.7	1.16

## Data Availability

The data will be available in the DOCTA repository of the Complutense University of Madrid.

## Supplementary Materials

The following supporting information can be downloaded at: https://www.mdpi.com/article/10.3390/jfb16110406/s1, Figure S1: BSE cross-sectional micrographs of PEO/PCL system after four days of immersion in modified α-MEM solution at 37 °C, showing regions with partial (left) and complete (right) hydration of the PEO layer. The table provides EDS analysis in the specified locations. Note the low amount of Cl at all locations, despite the obvious presence of a crevice. Figure S2: Micro-CT 3D video of specimens after 4 days of immersion: (a,b) Mg pure and (c,d) PEO coating. Two different viewing projections are given per sample.
