# Supplementary material for: Corrosion-Modulating Effect of Pharmaceutical Agents in a Hybrid Coating System on Pure Magnesium
Source: J Funct Biomater. 2025 Oct 30;16(11):406. doi: 10.3390/jfb16110406 (PMC12653425; doi:10.3390/jfb16110406)
Supplement: Supplementary file 1 [file jfb-16-00406-s001.zip › jfb-3930349-supplementary.pdf]

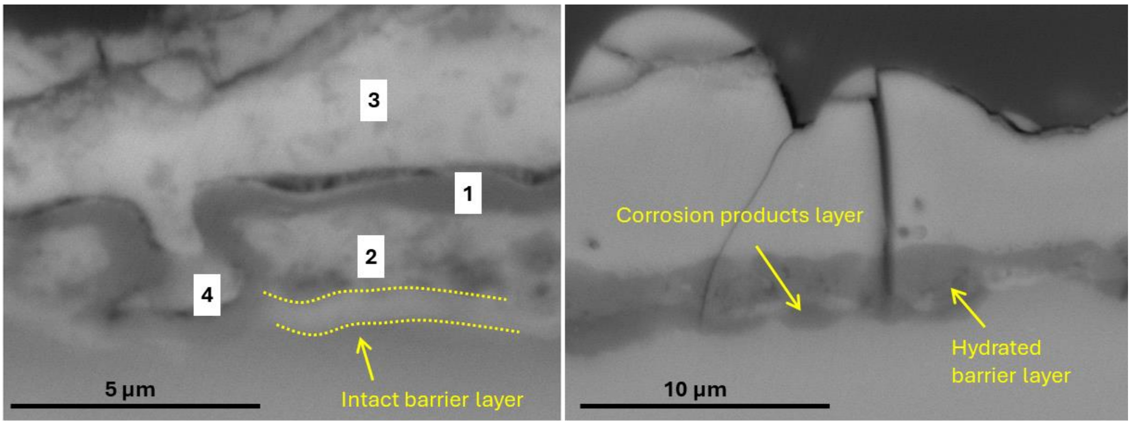

Position/EDS (at. %)	O	Mg	Si	P	Cl	Ca
1	61.24	14.94	5.63	7.17	0.06	10.96
2	59.24	29.59	7.61	2.24	0.05	1.27
3	64.40	4.89	0.25	11.94	0.11	18.41
4	54.31	28.66	5.24	4.86	0.12	6.81

Supplementary Figure 1. BSE cross-sectional micrographs of PEO/PCL system after 4 days of immersion in modified α-MEM solution at 37 ^{o}C, showing regions with partial (left) and complete (right) hydration of the PEO layer. The table provides EDS analysis in the specified locations. Note the low amount of Cl at all locations, despite the obvious presence of crevice.

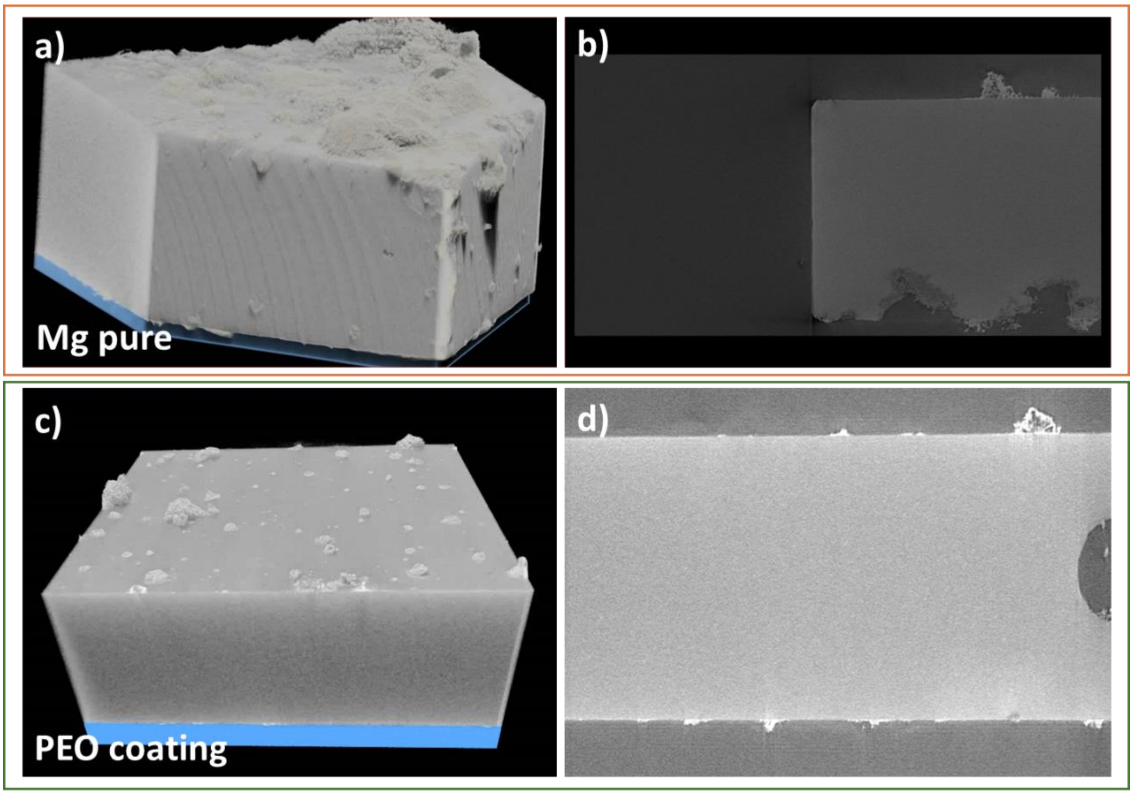

Supplementary Figure 2. Micro-CT 3D video of specimens after 4 days of immersion: (a,b) Mg pure and (c,d) PEO coating. Two different viewing projections are given per sample.
